# Genome-Wide Identification and Characterization of *BrrTCP* Transcription Factors in *Brassica rapa ssp. rapa*

**DOI:** 10.3389/fpls.2017.01588

**Published:** 2017-09-12

**Authors:** Jiancan Du, Simin Hu, Qin Yu, Chongde Wang, Yunqiang Yang, Hang Sun, Yongping Yang, Xudong Sun

**Affiliations:** ^1^Key Laboratory for Plant Diversity and Biogeography of East Asia, Kunming Institute of Botany, Chinese Academy of Sciences Kunming, China; ^2^Germplasm Bank of Wild Species, Kunming Institute of Botany, Chinese Academy of Sciences Kunming, China; ^3^University of Chinese Academy of Sciences Beijing, China; ^4^College of Plant Protection, Yunnan Agricultural University Kunming, China

**Keywords:** TCP, transcription factors, expression analysis, turnip, cell proliferation

## Abstract

The teosinte branched1/cycloidea/proliferating cell factor (TCP) gene family is a plant-specific transcription factor that participates in the control of plant development by regulating cell proliferation. However, no report is currently available about this gene family in turnips (*Brassica rapa ssp. rapa*). In this study, a genome-wide analysis of TCP genes was performed in turnips. Thirty-nine *TCP* genes in turnip genome were identified and distributed on 10 chromosomes. Phylogenetic analysis clearly showed that the family was classified as two clades: class I and class II. Gene structure and conserved motif analysis showed that the same clade genes have similar gene structures and conserved motifs. The expression profiles of 39 *TCP* genes were determined through quantitative real-time PCR. Most CIN-type *BrrTCP* genes were highly expressed in leaf. The members of CYC/TB1 subclade are highly expressed in flower bud and weakly expressed in root. By contrast, class I clade showed more widespread but less tissue-specific expression patterns. Yeast two-hybrid data show that BrrTCP proteins preferentially formed heterodimers. The function of *BrrTCP2* was confirmed through ectopic expression of *BrrTCP2* in wild-type and loss-of-function ortholog mutant of Arabidopsis. Overexpression of *BrrTCP2* in wild-type *Arabidopsis* resulted in the diminished leaf size. Overexpression of *BrrTCP2* in triple mutants of *tcp2/4/10* restored the leaf phenotype of *tcp2/4/10* to the phenotype of wild type. The comprehensive analysis of turnip TCP gene family provided the foundation to further study the roles of TCP genes in turnips.

## Introduction

Teosinte branched1/cycloidea/proliferating cell factor (TCP) gene family is a plant-specific transcription factor that regulates plant growth by controlling cell proliferation. TCP gene family has been identified in many plant species. For instance, *Arabidopsis* has 24 TCP genes, *Oryza sativa* has 28 *TCP* genes, tomato has 30 *TCP* genes, *Populus euphratica* has 33 *TCP* genes, *Populus trichocarpa* has 36 *TCP* genes, *Citrullus lanatus* has 27 *TCP* genes, and *Prunus mume* has 19 *TCP* genes (Martin-Trillo and Cubas, 2010; Parapunova et al., 2014; Ma X. et al., 2016; Shi et al., 2016; Zhou et al., 2016). The TCP domain contains a 59-amino-acid basic helix–loop–helix (bHLH) motif involved in DNA binding and protein–protein interaction (Martin-Trillo and Cubas, 2010). On the basis of the TCP domains, the members of the TCP family can be grouped into two subfamilies: class I (PCF or TCP-P class) and class II (TCP-C class) (Kosugi and Ohashi, 2002; Navaud et al., 2007; Martin-Trillo and Cubas, 2010). The difference between the two is a four-amino-acid deletion in the TCP domain in class I compared with class II.

Class I TCP genes are assumed to stimulate cell proliferation and leaf development, based mainly on the expression of rice *PCF1/PCF2* and *AtTCP20* in meristematic tissuses (Kosugi and Ohashi, 1997; Li et al., 2005). In *Arabidopsis*, loss-of-function *tcp15* mutant did not show any significant differences in comparison with wild-type plants. TCP15 fusion with SRDX repression domain elucidated that TCP15 regulated plant development via auxin response (Uberti-Manassero et al., 2012). *tcp14 tcp15* double mutants displayed shortened internode length, altered leaf shape, and severe reduction in seed germination capability compared with wild type (Kieffer et al., 2011; Resentini et al., 2015). Moreover, AtTCP9 acts repeatedly with AtTCP20 in regulating leaf senescence via the jasmonate signaling pathway (Danisman et al., 2012). However, pentuple mutant *tcp8 tcp15 tcp21 tcp22 tcp23* exhibited upregulated expression levels of *SHOOT-MERISTEMLESS, BP*, and *CYCA1:1* and resulted in large leaf blades (Aguilar Martinez and Sinha, 2013).

Class II can be further divided into subclades: CIN and CYC/TB1 (Martin-Trillo and Cubas, 2010). Class II usually prevented cell proliferation and differentiation during the development of leaf blades. In *Arabidopsis*, CIN-type genes *TCP2, TCP3, TCP4, TCP10*, and *TCP24* are targets of miR319a. *jaw-D* (overexpression of miR319a) plants resulted in large and crinkled leaves (Palatnik et al., 2003). Single loss-of-function miR319a-targeted *TCPs* had slight developmental phenotypes. Double mutants (*tcp2 tcp4*) and triple mutants (*tcp2 tcp4 tcp10*) showed less increase in leaf size with some crinkled signs than *jaw-D* plants. miR319a-targeted TCP transcription factors negatively regulated leaf growth and positively regulated leaf senescence via mediating *LOX2* gene expression (Schommer et al., 2008). miR319a-targeted *TCP4* is required for proper petal growth and development (Nag et al., 2009). miR319a-targeted TCPs interact with ASYMMETRIC LEAVES2 and ensure normal leaf development by repressing the expression of *BP* and *KNAT2* by binding their promoter (Li Z. et al., 2012).

Turnip (*Brassica rapa ssp. rapa*) is one of the most economically important vegetable crops in the Tibet Plateau. However, no report on the turnip (*Brassica rapa ssp. rapa*) TCP family exists. The turnip genome has been sequenced and assembled (Lin et al., 2014), providing the basis for determining the turnip family. In this study, genome-wide identification of TCP transcription factors in turnips is performed. Thirty-nine *BrrTCP* genes were identified in the turnip genome, and their phylogenetic relationship, gene structure, protein motifs, chromosome location, transcript levels in different tissue, and forms of homo- and heterodimer interaction were analyzed. Furthermore, a CIN-type gene, *BrrTCP2*, was functionally characterized in transgenic wild-type and loss-of-function mutant *Arabidopsis*. Our findings indicate that the BrrTCP2 plays a vital function in leaf development by modulating cell division.

## Materials and methods

### Identification of the *TCP* genes in turnips

The genome sequence of turnips was downloaded from www.bioinformatics.nl/brassica/turnip. To find all *TCP* genes in turnips, NCBI BLASTn searches against a local database built using nucleic acid sequences were performed using sequences from all 24 known *TCPs* from *Arabidopsis*. Subsequently, the Pfam database was used to determine if each candidate *TCP* sequence was a member of the *TCP* gene family. To exclude overlapping genes, all candidate *TCP* genes were aligned using DNAMAN 4.0 (Lynnon Biosoft) and checked manually. All nonoverlapping *TCP* genes were used for further analysis.

### Analysis of conserved motifs

Conserved motifs of BrrTCP proteins were analyzed using MEME (http://meme-suite.org/tools/meme) with the following parameters: (1) the optimum motif width was set from 6 to 200, and (2) the maximum number of motifs was set to identify 20 motifs.

### Gene structure, genomic distribution, and divergence time estimation of *BrrTCP* genes

*BrrTCP* genes were mapped on chromosomes by confirming their detailed chromosomal positions supplied by the Turnip Genome Database. To illustrate the structure of introns and exons of *BrrTCP* genes, full-length genome and coding sequences of *BrrTCP* genes were subjected to online GSDS analysis (http://gsds.cbi.pku.edu.cn/). To determine their physical location, the starting positions of all *BrrTCP* genes on each chromosome were confirmed based on a local database of the complete sequence of the turnip genome through BLASTn searching. The segmental and tandem duplication regions were obtained from MCscanX. For synteny analysis, synteny block of the turnip gene was visualized using Circos (http://circos.ca/). Synonymous (Ks) and nonsynonymous (Ka) substitution rates were estimated by the codeml program of PAML4 (Yang, 2007). The divergence time (T) of turnip gene pairs was calculated using the formula *T* = Ks/2λ, where λ represents the divergence rate of 1.5 × 10^−8^ for *Arabidopsis* (Gaut et al., 1996).

### Plant material and growth conditions

*Brassica rapa ssp. rapa* “KTRG-B48a” from Xianggelila City, Yunnan Province, China, was used. For root collection, seedlings were grown on a Whatman filter paper and watered with 1/2 MS medium for 2 weeks. For other tissues, plants were grown in the greenhouse (22°C) under long-day conditions (16 h light/day).

### Quantitative RT-PCR

Total RNA was extracted using Eastep™ Universal RNA Extraction Kit (Promega, Shanghai, China) from roots of 1-week-old plants, leaves and stems of 8-week-old plants, and floral buds of 10-week-old plants. RNA quality and concentration were assessed using electrophoresis and ND-1000 Spectrophotometer (NanoDrop Technologies, Delaware, USA). Two micrograms of total RNA were reverse transcribed using GoScript™ Reverse Transcription System (Promega). Quantitative RT-PCR (qRT-PCR) was performed with ABI7500 Real-Time PCR System using TransStart® Top Green qPCR SuperMix (TransGen, Beijing, China). *BrrACT2* was used as reference gene. The primers are listed in Table S1. Triplet biological replicates were analyzed.

### Yeast two-hybrid assays

All *BrrTCP* ORFs were amplified from seedling cDNA of KTRG-B48a into pENTR vector used primers (Table S2). The entry vectors were subcloned into the pGADT7 prey vector (pDEST-GSDT7) and pGBKT7 bait vector (pDEST-GBKT7) using gateway method according to Bai et al. (2016). The prey vector was transformed into yeast strain Y187, and all bait vectors were transformed into yeast strain Y2H gold and selected on SD plates lacking Leu and Trp. After selection, the yeasts harboring prey and bait plasmids were spotted onto control medium (SD plates lacking Leu and Trp) and test medium (SD plates lacking Leu, Trp, and His). Yeast growth was observed daily in a growth chamber at 28°C for 2–5 days.

### Laser confocal microscopy

The entry vectors of *BrrTCP* were subcloned into *pRI101-GFP* using gateway method. *35S:GFP-TCPs* were introduced into *Agrobacterium* GV3101. Positive transformants were selected on LB agar medium (50 mg/L kanamycin, 50 mg/L gentamicin, and 50 mg/L rifampicin). After 3 days, a single colony was inoculated into 2 mL LB liquid medium. Target inserts were confirmed through PCR. *Nicotiana benthamiana* plants were grown at 22°C under 16 h light/8 h dark conditions. One-month-old plants were used for infiltration. Two days before infiltration, 2 mL cultures of the *Agrobacterium* strains were inoculated from single colonies on plates and grown for 24 h at 28°C. The working cultures were inoculated from the starter culture at a 1:100 ratio. Cells were harvested through centrifugation at 3,000 *g*, 22°C, for 5 min. Cell pellets were resuspended in infiltration medium (10 mM MgCl_2_; 10 mM MES, pH 5.7; and 150 μM acetosyringone) with OD600 adjusted to 1. Resuspended cell cultures were kept at room temperature for 3 h before infiltration. Leaf infiltration was conducted by depressing a 1 mL disposable syringe to the surface of fully expanded leaves and slowly depressing the plunger. Leaf discs were collected 72 h after infiltration for measurements using green fluorescent protein (GFP) fluorescence assay. Fluorescence images were obtained under an Olympus FV1000 laser confocal microscope (Olympus, Tokyo, Japan), with excitation at 488 nm and emission at 505–530 nm.

### Transformation of *Arabidopsis*

The *35S:GFP-BrrTCP2* construct was introduced into *Agrobacterium* GV3101, which was used to transform wild-type *Arabidopsis* via floral dip (Clough and Bent, 1998). T0 seeds were screened on MS medium containing 30 mg L^−1^ kanamycin. All transgenic lines overexpressing *35S:GFP-BrrTCP2* were verified by Western blot using GFP antibody. *OXBrrTCP2* x tcp2/4/10 plants were obtained by crossing *OXBrrTCP2* with *tcp2/4/10*.

### Scanning electron microscopy

The sixth rosette leaves were selected for scanning electron microscopic analysis as previously described by Sun et al. (2013). The samples were observed and photographed under a scanning electron microscope (Gemini Sigma 300/VP SEM, Carl Zeiss, Germany) at an accelerating voltage of 10 kV.

## Results

### Identification of *TCP* genes in turnips

**Figure 1:**
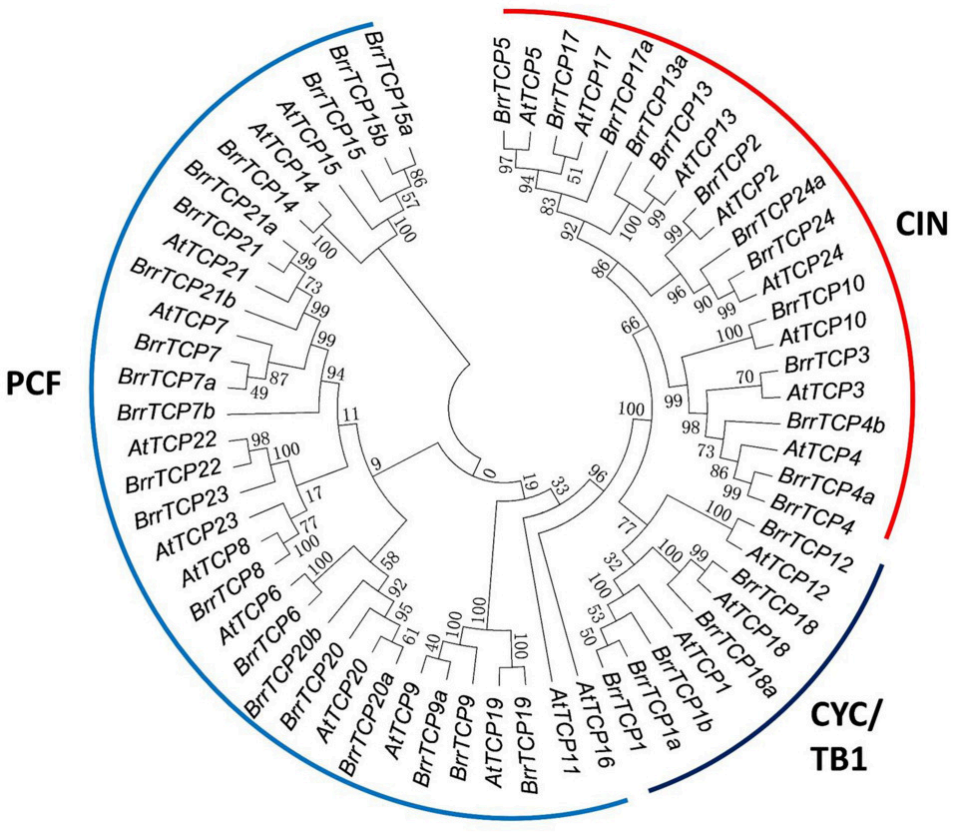
Phylogenetic tree of TCP proteins from turnips and *Arabidopsis*. The phylogenetic tree was generated using the neighbor-joining (NJ) method implemented in MEGA 7.0 software with JTT model and pairwise gap deletion option. Bootstrap analysis was conducted with 1,000 iterations.

The release of the complete turnip genome sequences allowed the genome-wide identification of turnip genes (Lin et al., 2014). In the present study, BLAST was carried out to search *BrrTCP* in the turnip genome. The obtained sequences were further verified through hidden Markov model. Finally, a total of 39 nonredundant BrrTCPs were identified from turnip genome. The BrrTCP genes were named following the nomenclature of *Arabidopsis thaliana* depending on protein sequence similarities (Figure 1). Sequence analysis revealed that AtTCP11 and 16 had no orthologs in turnip. AtTCP1, 4, 7, 9, 13, 15, 17, 18, 20, 21, and 24 had more than one ortholog in the turnip genome. Given the lack of standard annotations assigned to the 39 TCPs in turnips, the orthologs were designated as shown in Table 1 based on protein sequence similarities to their orthologs in *Arabidopsis*.

**Table 1:** TCP gene family in turnip.

**Gene Name**	**Accession number**	**Length(aa)**	**MW(Da)**	**pI**	**Chr. Location**
BrrTCPl	KY607998	351	39,826.39	6.47	Chr07: 17379980–17381035
BrrTCPla	KY607999	347	39,385.23	6.55	Chr07: 16762673–16763813
BrrTCPlb	KY608000	346	39,314.74	5.54	Chr02: 10171599–10172639
BrrTCP2	KY608001	384	42,403.17	7.47	Chr03: 23210500–23211639
BrrTCPS	KY608002	341	37,222.15	7.53	Chr08: 1024759–1025793
BrrTCP4	KY608003	406	44,173.60	7.34	Chr05: 20022294–20023514
BrrTCP4a	KY608004	407	44,425.04	8.13	Chr03: 17113530–17114753
BrrTCP4b	KY608005	348	38,161.10	7.06	Chr01: 24280209–24281261
BrrTCP5	KY608006	366	40,706.24	6.52	Chr03: 21107231–21108331
BrrTCP6	KY608007	224	24,641.60	8.02	Chr04: 8105848–8106522
BrrTCP7	KY608008	252	27,180.36	9.25	Chr06: 16403133–16407846
BrrTCP7a	KY608009	252	18,681.04	8.06	Chr02: 25460706–25461236
BrrTCP7b	KY608010	219	23,770.77	8.21	Chr01: 8925053–8929517
BrrTCP8	KY608011	394	41,385.39	6.38	Chr09: 9571412–9572956
BrrTCP9	KY608012	325	34,463.24	9.68	Chr05: 2500244–2501218
BrrTCP9a	KY608013	318	33,838.78	9.88	Chr03: 10874971–10875906
BrrTCPl0	KY608014	348	38,671.40	6.71	Chr05: 7091828–7092874
BrrTCPl2	KY608015	336	38,324.79	8.66	Chr02: 11017245–11018356
BrrTCP13	KY608016	320	35,694.44	7.07	Chr03: 14413642–14414472
BrrTCP13a	KY608017	309	34,451.40	7.56	Chr05: 23621188–23622581
BrrTCP14	KY608018	466	50,250.46	6.83	Chr06: 10402184–10403578
BrrTCPl5	KY608019	321	34,030.17	7.26	Chr07: 18427674–18428612
BrrTCP15a	KY608020	245	25,663.89	6.67	Chr07: 16070535–16071269
BrrTCPl5b	KY608021	246	25,691.98	6.74	Chr02: 11422832–11423572
BrrTCP17	KY608022	301	33,762.21	6.83	Chr02: 25620883–25621956
BrrTCP17a	KY608023	246	27,372.44	6.82	Chr03: 1428184–1428909
BrrTCP18	KY608024	424	48,340.37	8.69	Chr03: 17927181–17929425
BrrTCP18a	KY608025	285	32,645.80	9.46	Chr01: 22021125–22022887
BrrTCP19	KY608026	281	30,182.56	5.52	Chr02: 8815586–8816431
BrrTCP20	KY608027	200	22,050.51	5.48	Chr02: 22055944–22058191
BrrTCP20a	KY608028	311	32,789.26	7.68	Chr06: 22527874–22528797
BrrTCP20b	KY608029	278	29,658.68	6.38	Chr09: 1164212–1165048
BrrTCP21	KY608030	234	24,508.21	8.81	Chr10: 16190790–1619500
BrrTCP21a	KY608031	234	24,261.29	10.20	Chr03: 1493264–1493968
BrrTCP21b	KY608032	229	23,872.73	9.55	Chr02: 2637716–2638405
BrrTCP22	KY608033	374	39,116.38	8.95	Chr02: 12405545–12406669
BrTCP23	KY608034	356	37,152.33	7.64	Chr07: 19406027–19407097
BrrTCP24	KY608035	319	35,402.64	7.93	Chr09: 22077911–22078867
BrrTCP24a	KY608036	313	34,970.16	7.44	Chr08: 16381466–16383191

**Figure 2:**
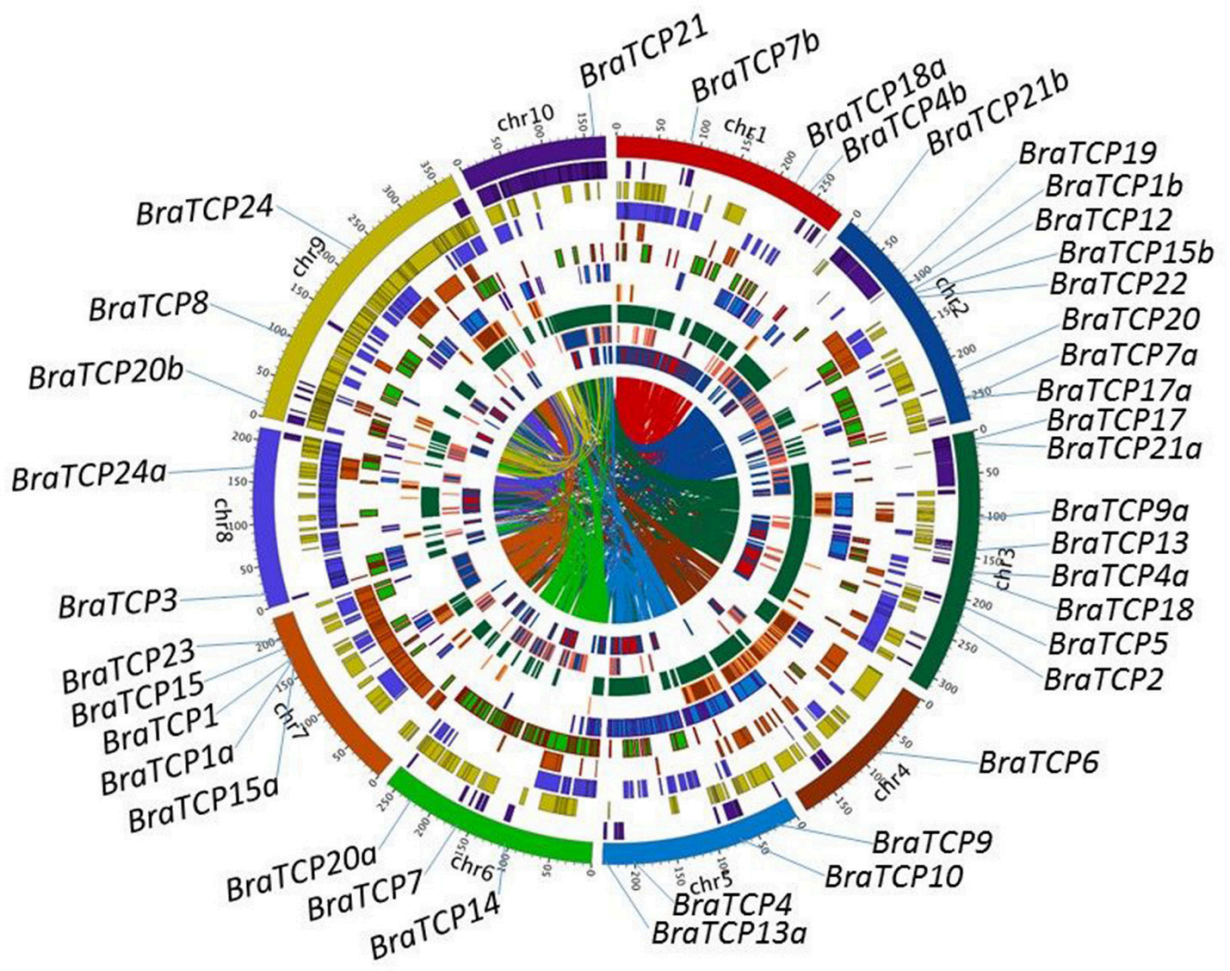
BrrTCPs' chromosome distributions, synteny block, and the turnip genome duplication event caused paralogous relationships. Chromosomes are shown in different colors and in the outer circle, where the numbers represent the chromosome length in 100 Kb. The BrrTCP genes are marked on the approximate positions with specific color lines on the circle. Filled blocks in different colors denote the syntenic relationships of turnip TCP genes.

Information of the *BrrTCP* gene family members is shown in Table 1. The ORF length of *BrrTCP* genes varied from 603 to 1,401 bp, encoding polypeptides of 200–466 amino acids, with a predicted molecular mass of 22.05–50.25 kDa. The theoretical pI ranged from 5.48 to 10.20. Genomic localization of each *BrrTCP* in turnips is shown in Figure 2. These genes were distributed in all 10 turnip chromosomes. The maximum number of *BrrTCP* genes per chromosome was found for chromosome A02 with 9 *TCP* genes. Eight genes were located at chromosome 3, and five and four genes were located at chromosomes 7 and 5, respectively. Chromosomes 4 and 10 had the minimum *BrrTCP* genes, only one each. Three genes each were located on chromosomes 1, 6, and 9. Chromosome 8 contained two *BrrTCP* genes. A total of seven pairs of putative BrrTCP paralog proteins were produced by segmental duplication, which were distributed in different chromosomes. These results indicated the large-scale segmental duplication events involved in the expansion of BrrTCP gene family in turnips.

The divergence time (T) of seven pairs of BrrTCP paralog proteins was estimated by measuring the synonymous (Ks) and nonsynonymous (Ka) mutation rates (Table 2). The estimated divergence time (T) for the BrrTCP paralogs was from 10.3067 to 27.8600 million years ago (MYA), with an average duplication time of approximately 18.7862 MYA. Schranz et al. (2006) estimated the time of very early radiation of Brassicaceae at 34 MYA. Comparative physical mapping studies have confirmed genome triplication in a common ancestor of *B. oleracea* (O'Neill and Bancroft, 2000) and *B. rapa* (Park et al., 2005) since its divergence from the *A. thaliana* lineage at least 13–17 MYA (Yang et al., 1999; Town et al., 2006; Beilstein et al., 2010). The divergence time of three pairs of BrrTCP paralogs (*BrrTCP9*/*9a, BrrTCP22*/*23*, and *BrrTCP24*/*24a*) occurred precedent to the period of the origin of the *B. rapa*. The ω values for the BrrTCP paralogs were less than one. However, two pairs of BrrTCPs (BrrTCP1a/1b, ω = 0.3794; BrrTCP24/24a, ω = 0.3646) achieved relatively large ω values, which suggested that they may have evolved rapidly from those of the last common ancestor.

**Table 2:** Dates of duplication of duplicated gene pairs.

**Seql**	**Seq2**	**Identity (%)**	**Ks**	**Ka**	**to**	**T (MYA)**
BrrTCPla	BrrTCPlb	73.24	0.3092	0.1173	0.379366106	10.3067
BrrTCP4	BrrTCP4a	79.24	0.416	0.0443	0.106490385	13.8667
BrrTCP9	BrrTCP9a	70.76	0.6658	0.1743	0.261790327	22.1933
BrrTCPl5a	BrrTCPl5b	88.10	0.3792	0.0404	0.106540084	23.8867
BrrTCP2l	BrrTCP2la	86.50	0.544	0.0441	0.081066176	18.1333
BrrTCP22	BrrTCP23	71.76	0.4577	0.1117	0.244046319	15.2567
BrrTCP24	BrrTCP24a	68.13	0.8358	0.3047	0.3645609	27.8600

### Gene structure and conserved motifs

**Figure 3:**
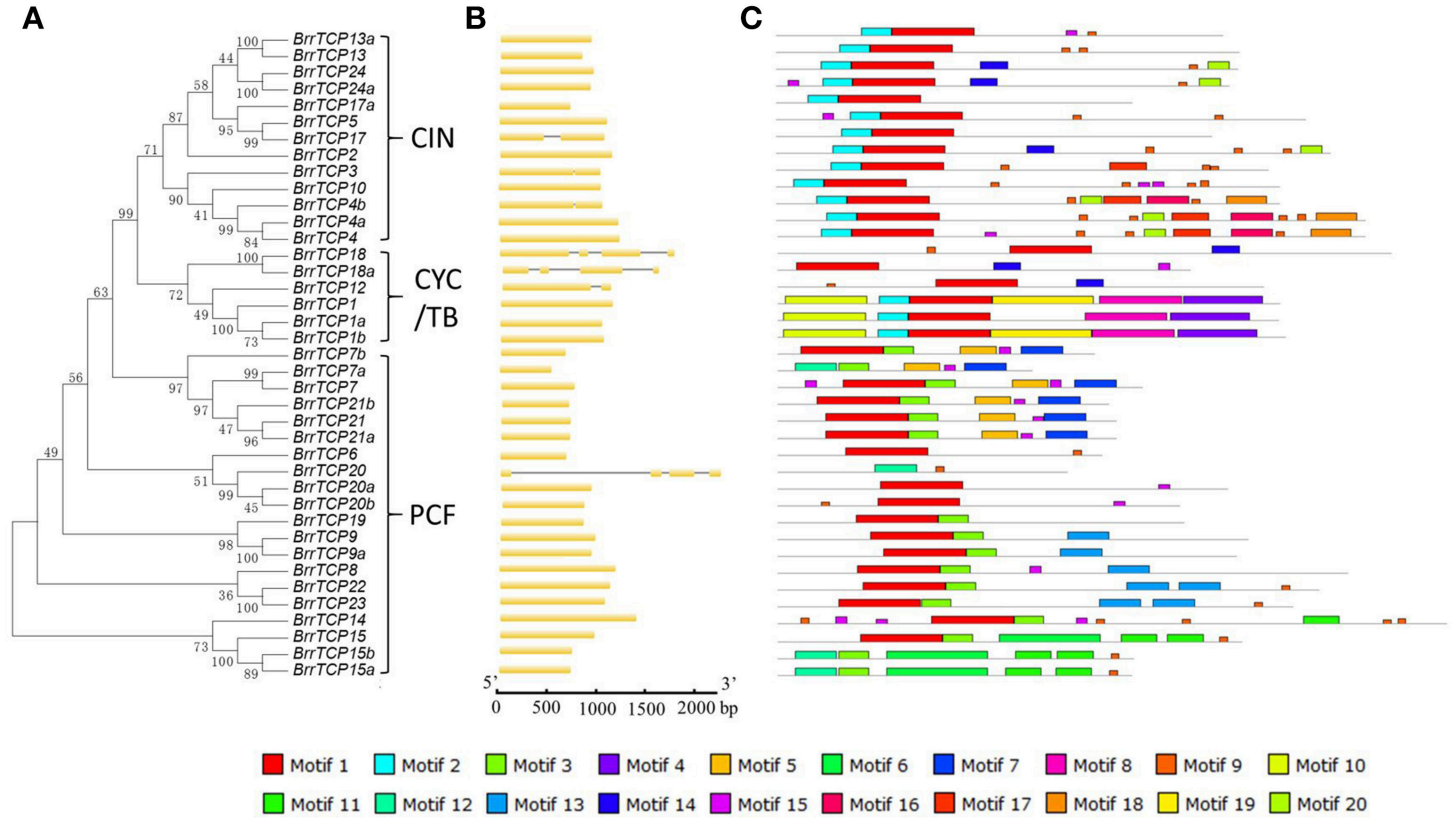
Genomic structure and motif composition of turnip TCPs. **(A)** Phylogenetic tree of turnip TCP proteins. **(B)** Genomic structure of turnip gene. Exons and introns are represented with yellow boxes and black lines. **(C)** The conserved motifs in turnip TCP proteins were identified using MEME. Each motif is represented with a specific color.

To better understand the diversification of the TCP genes in turnips, the exon/intron organization and conserved motifs of BrrTCPs were analyzed. A new neighbor-joining phylogenetic tree was constructed using the protein sequences of BrrTCPs (Figure 3A). The genome sequences and corresponding coding sequences of *TCP* genes in turnip analysis revealed that most *BrrTCP* genes have similar gene structures in the same group (Figure 3B). A total of 32 members of *BrrTCP* gene family have one exon (82%), 4 genes have two exons (10%), and 3 have four exons (8%). In *Arabidopsis*, the number of exons ranged from one to four, and 82% of the genes contained only one exon. The *TCP* genes in turnips exhibited similar gene structure. The MEME online tool was used to predict the conversed motif composition of BrrTCPs (Figure 3C). The number of motifs varied from 2 to 11. The motifs were evaluated using InterProScan for annotation. The results revealed that motifs 1, 2, and 3 were identified as TCP domain. In addition, some motifs only presented at specific subclades, such as motifs 6, 7, 10, and 19 in BrrTCP1, 1a, and 1b; motif 9 in BrrTCP15, 15a, and 15b; and motif 16 in BrrTCP4, 4a, and 4b, suggesting that they may have subclade-specific functions.

**Figure 4:**
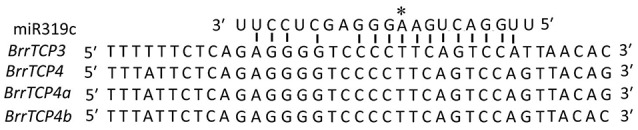
Alignment of putative target areas for miR319c (aligned in reverse). The “^*^” and above sequences indicate the cleavage site.

In *Arabidopsis*, miR319 controls jasmonate biosynthesis and senescence by cleaving *TCP* transcription factors (Schommer et al., 2008) and petal development (Nag et al., 2009). The AtmiR319-targeted *TCP* genes, namely, *AtTCP2, AtTCP3, AtTCP4, AtTCP10*, and *AtTCP24*, all belong to the CIN clade. Similarly, four *TCP* genes in turnips contain miR319 binding sites (Figure 4), and all of them were CIN family members.

### Subcellular localization

**Figure 5:**
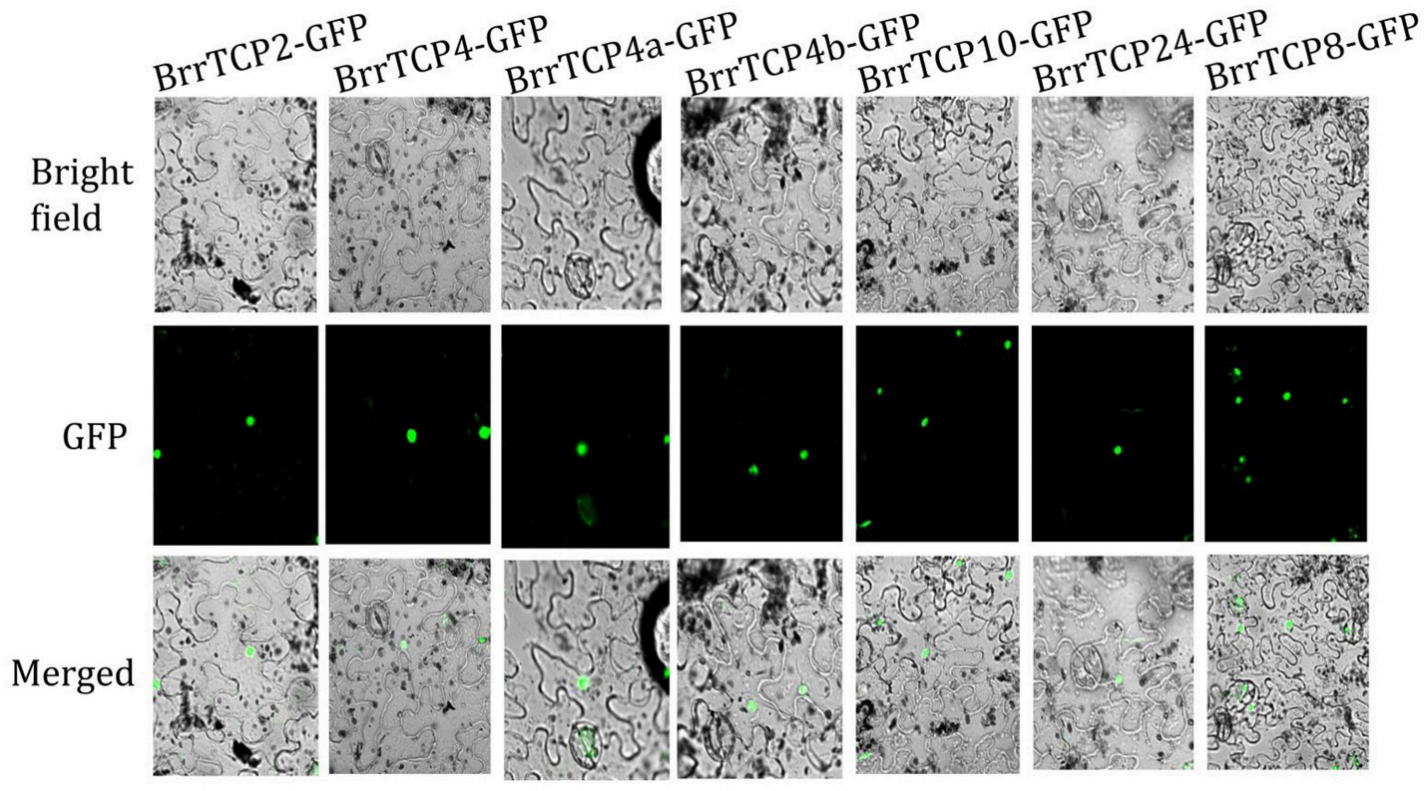
Subcellular localization of 35S:BrrTCP-GFP in *Nicotiana benthamiana* leaves. BrrTCP-GFP localized in the nucleus.

The known members of the TCP gene family function as transcription factors. The GFP gene was fused with *BrrTCPs* as a reporter. The GFP signals of BrrTCP-GFPs were detected in the nucleus using laser confocal microscopy (Figure 5), which suggested that BrrTCPs functioned as transcription factors.

### qRT-PCR analysis of the turnip *TCP* genes

**Figure 6:**
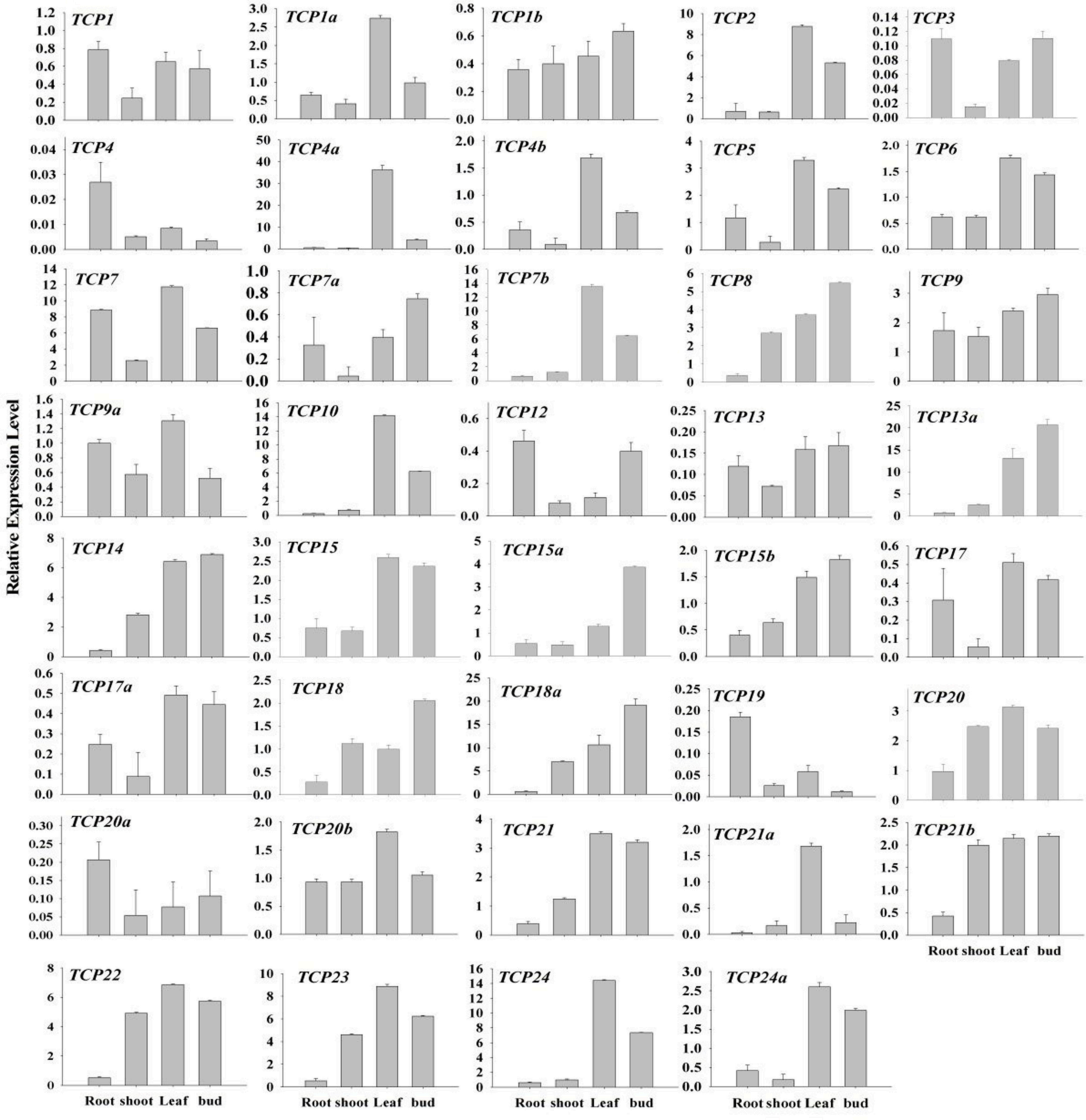
Expression patterns of turnip *TCP* genes in different tissues. The expression profile data of *BrrTCP* genes in roots, shoots, leaves, and buds were obtained through qRT-PCR.

The expression pattern of a gene is always relative to its function (Xu et al., 2015). To probe possible functions of *BrrTCP* genes in turnips, qRT-PCR was performed to examine the expressions of *BrrTCP* genes in different organs of turnips (Figure 6). Interestingly, expression levels of most CIN-type *BrrTCP* genes were high in leaves and weak in the roots and stems. *BrrTCP18* and *BrrTCP18a*, the members of CYC/TB1 subclade, are highly expressed in flower buds and weakly expressed in roots. In contrast, class I clade showed more widespread but less tissue-specific expression patterns; for example, *BrrTCP7* and *BrrTCP9* are highly expressed in roots, stems, leaves, and flower buds; *BrrTCP8, 14, 18a, 20, 21b, 22*, and *23* are highly expressed in the stems, leaves, and flower buds; and *BrrTCP7b, 13a, 15*, and *21* are highly expressed in leaves and flower buds. These results indicated that every clade possessed a characteristic expression profile.

### Interactions between turnip TCP proteins

**Figure 7:**
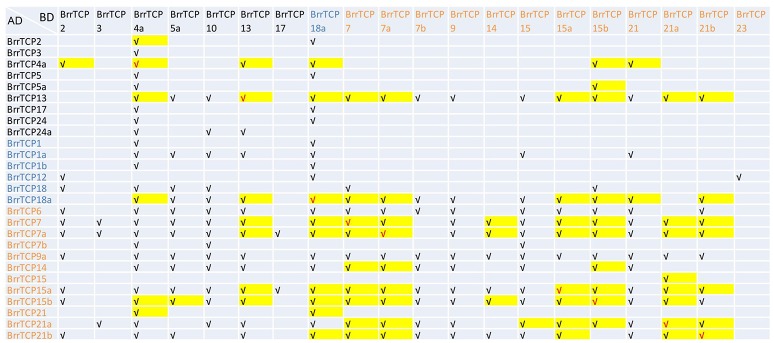
Interaction of BrrTCP proteins in yeast two-hybrid assay. AD-fusion is listed in the left column. BrrTCP protein names are ordered according to their subclades (CIN subclade is represented by black, TB1 by blue, and PCF by red).

TCP proteins tend to form homodimers or heterodimers with other TCP proteins, and dimerization may be required for their DNA-binding activity and hence for their biological activity. Yeast two-hybrid screening was carried out to investigate the interactions among the BrrTCP proteins, as shown in Figure 7, where the proteins are arranged according to their subclades except their autoactivation activity. A total of 8 out of 39 AD-fusion proteins tested had autoactivation activity in yeast. Meanwhile, out of the 39 BD-fusion proteins tested, 13 had autoactivation activity in yeast. Among them, five BrrTCP proteins had autoactivation activity in AD- and BD-fusion proteins. Although we selected 219 interactions, this number may not be accurate due to autoactivation. A total of 90 (45 pairs) proteins interacted in AD- and BD-orientations, including 9 homodimer formations. The class I BrrTCP transcription factors formed 91 homo- or heterodimers within class I members. Meanwhile, class II BrrTCP transcription factors formed 42 homo- or heterodimers within class II. The members of class I proteins more preferentially formed dimerization properties in the same class than class II.

### Overexpression of *BrrTCP2* rescued the *tcp2/4/10* phenotype

**Figure 8:**
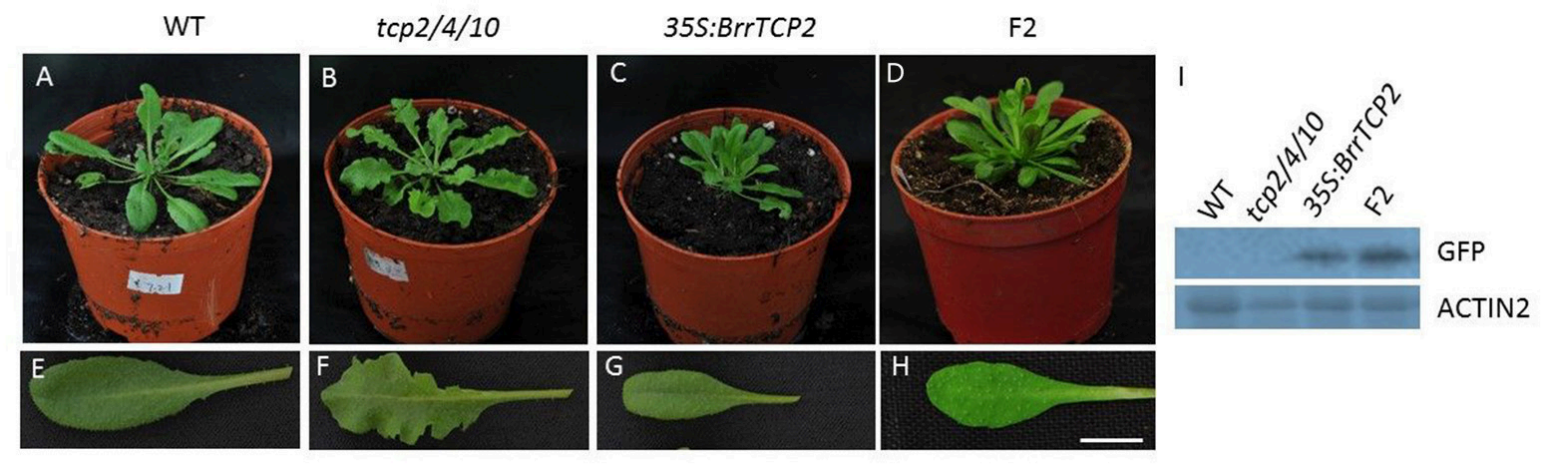
Phenotype effects of constitutive expression of *BrrTCP2* in transgenic *Arabidopsis*. **(A,E)** Phenotype of wild-type *Arabidopsis*. **(B,F)** Phenotype of *tcp2/4/10* mutants. **(C,G)** Expression of *BrrTCP2* in wild-type *Arabidopsis*. **(D,H)** Phenotype of the homozygote F2 plants alleviates the phenotype of the *tcp2/4/10* mutants. **(I)** Western blot analysis of BrrTCP2 protein levels in transgenic plants. BrrTCP2 protein was analyzed through immunoblotting using an anti-GFP antibody. AtACT2 served as the control. Bar = 0.5 cm.

**Figure 9:**
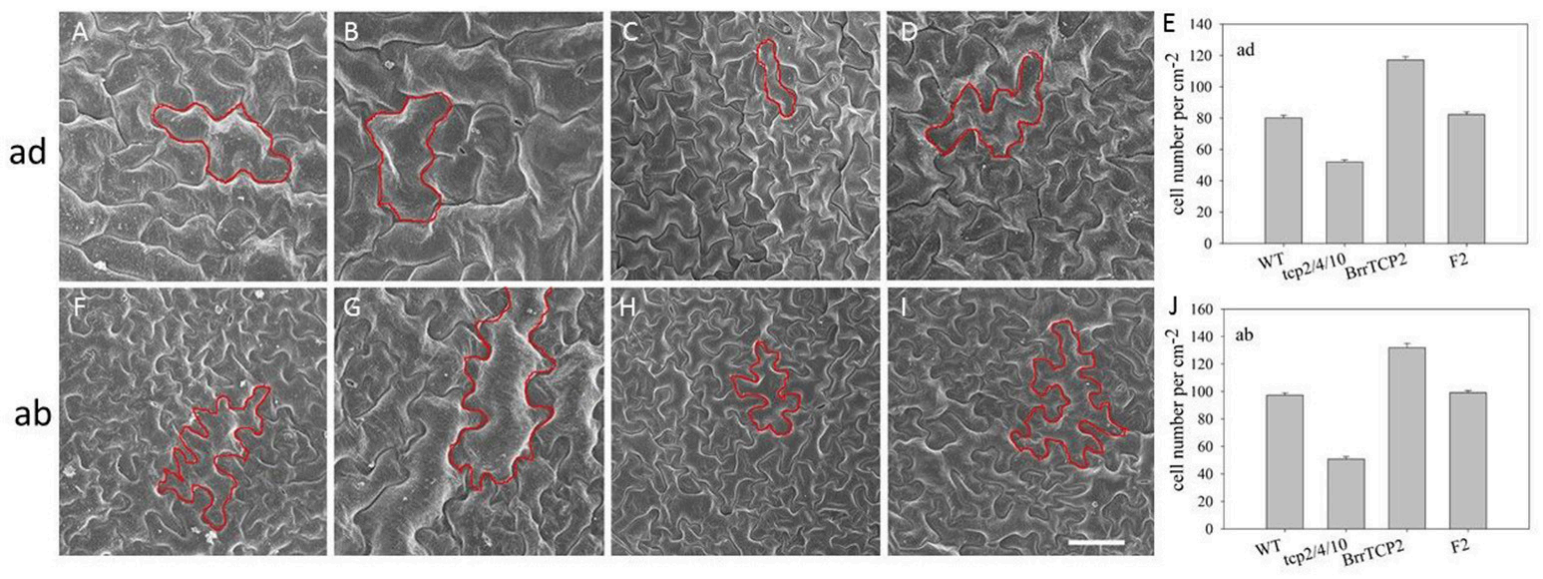
Scanning electron micrographs of leaf epidermal cells. Adaxial epidermis in the sixth rosette leaf from WT **(A)**, *tcp2/4/10* **(B)**, *35S:BrrTCP2* transgenic plants **(C)**, and F2 **(D)**. **(E)** Number of adaxial epidermis cells of sixth rosette leaves. Abaxial epidermis in the sixth rosette leaf from WT **(F)**, *tcp2/4/10* **(G)**, *35S:BrrTCP2* transgenic plants **(H)**, and F2 **(I)**. **(J)** Number of abaxial epidermis cells of sixth rosette leaves. Bar = 50 μm.

*BrrTCP2* is a member of the CIN subclade of TCP in turnips. Given the unavailability of *tcp*-related mutant in turnips, we constructed transgenic *Arabidopsis* plants overexpressing *BrrTCP2*. As shown in Figure 8, the sixth rosette leaf of *tcp2/4/10* triple mutants showed the most enhanced leaf size, with some signs of crinkling (Figures 8B,F). Meanwhile, the *OXBrrTCP2* plants showed the most diminished leaf size (Figures 8C,G). *OXBrrTCP2* line was crossed with *tcp2/4/10*, and the phenotype of the homozygote F2 plants restored the leaf phenotype of *tcp2/4/10* to the phenotype of wild type (Figures 8D,H). Western blot analysis showed that *OXBrrTCP2* and F2 plants had high expression levels, whereas no signal was detected in WT and *tcp2/4/10* plants (Figure 8I). *Arabidopsis* leaf size is normally regulated by the cell size and number. To assess the cell size and number, we selected a site midway along the length of the lamina and between the margin and the midvein of the expanded sixth rosette leaf for analysis using scanning electron microscopy. The adaxial epidermal cell size of *tcp2/4/10* was larger than WT (Figures 9A,B), and fewer cells were noted per unit area (Figure 9E). Meanwhile, the adaxial epidermal cell size of *OXBrrTCP2* plants was smaller than WT (Figures 9A,C), and more cells were noted per unit area (Figure 9E). The F2 plant had similar cell size and cell number per unit area with WT. The abaxial epidermal cell size and cell number were similar to the adaxial epidermis. The *tcp2/4/10* had larger cell size and fewer cell number per unit area than WT (Figures 9F,G,J), whereas *OXBrrTCP2* plants had smaller cell size and more cell number per unit area than WT (Figures 9F,H,J). Overexpression of *BrrTCP2* in *tcp2/4/10* also restored the abaxial epidermal phenotype of *tcp2/4/10* to WT (Figures 9D,I). *BrrTCP2* may have a function in cell division and/or differentiation.

## Discussion

TCP proteins are plant-specific transcription factors involved in the regulation of multiple processes during plant development and growth, such as leaf morphogenesis and senescence, flower development, circadian clock, endoreduplication, branching, fiber development, and phytohormone biosynthesis (Schommer et al., 2008; Nag et al., 2009; Sugio et al., 2011; Danisman et al., 2012; Hao et al., 2012; Li Z. Y. et al., 2012; Wang et al., 2013; Ma J. et al., 2016; Zhou et al., 2016). Previous studies revealed that all Brassicaceae, including *Arabidopsis* and *Brassica's*, underwent polyploidization events, such as γ triplication (135 MYA) and β (90–100 MYA) and α (24–40 MYA) duplications (Wang and Kole, 2015). *B. rapa* shares this complex history, with the addition of a whole-genome triplication (WGT) thought to have occurred between 13 and 17 million years ago (MYA) making “mesohexaploidy” a characteristic of the Brassiceae tribe of the Brassicaceae (Lysak et al., 2005). The Brassica genomes diploidized after this triplication event through genome fractionation and rearrangements (Mun et al., 2009). Several studies revealed that the three subgenomes did not behave similar and that the dominat subgenome retained most genes; in addition, the genome fractionation was not a random process, as certain gene families retained more copies (Park et al., 2005; Wang et al., 2011; Chalhoub et al., 2014). The BrrTCP gene family in turnips may be caused by genome duplication processes, including multiple segmental duplications, tandem duplication, transposition events, and whole-genome duplication. Except gene duplication, differences in exon/intron organizations can also clarify the evolutionary history of the gene family. The gene structure of *BrrTCPs* compared with the same clade showed that *TCP* genes shared similar exon/intron distribution in terms of exon length and intron numbers; meanwhile, BrrTCPs with the same clade displayed similar motif distribution. Similar to tomato TCP proteins (Parapunova et al., 2014), more interactions were found for class I proteins than class II proteins (91 vs. 42), although the number of interactions for class I and class II may be underestimated because of the autoactivating members. The interactions obtained by a comprehensive yeast two-hybrid screen of turnip TCP transcription factors have not yet been reported for TCP members from other species than tomato. Expression analysis and dimerization properties may help to identify TCP protein pairs that function together and explain observed functional redundancies in case of overlapping interaction maps of turnips in the future.

In *Arabidopsis, miR319*-targeted *AtTCP2, 3, 4, 10*, and 24 regulate leaf development and petal growth (Palatnik et al., 2003; Ori et al., 2007; Nag et al., 2009). The three closest turnip *TCP* genes have a putative binding site for *miR319c*. Gene function is also related to its expression profile (Zhou et al., 2016). In this study, we detected the expression patterns of 39 *BrrTCP* genes in four organs using qRT-PCR. These genes vary widely among the turnip organs. Two CIN-type genes (*BrrTCP4a* and *BrrTCP4b*), which are miR319c targeted, exhibited high expression levels in leaves, particularly *BrrTCP4a*. Meanwhile, *BrrTCP4* exhibited low expression levels in all detected organs. This phenomenon was also found in other duplicated gene pairs, such as *BrrTCP7, BrrTCP7a*, and *BrrTCP7b. BrrTCP7* showed high expression levels in roots, leaves, and flowers, whereas *BrrTCP7a* showed low expression levels in all detected organs. However, *BrrTCP7b* exhibited high expression levels in leaves and flowers. Gene duplication plays a vital role in the process of plant genomic and organismal evolution and confers new gene functions and the evolution of gene networks (Flagel and Wendel, 2009). Gene duplication might confer new functions to the paralogous *BrrTCP* genes. The other CIN-type genes, such as *BrrTCP2, BrrTCP10, BrrTCP13a*, and *BrrTCP24*, exhibited high expression levels not only in leaves but also in flowers. Turnip CIN-type genes may have a function in leaf and flower development. Class I and class II have antagonistic functions based on similar putative binding sites (Danisman et al., 2012). Class I *BrrTCP* genes, such as *BrrTCP7b* and *BrrTCP14*, were also detected to have high expression levels in leaves and flowers. In *Arabidopsis*, TCP14, which is homologous to BrrTCP14, acts repeatedly with TCP15 in modulating cell proliferation in developing leaf blades and flowers (Kieffer et al., 2011). Some members of class I and class II competitively regulated the cell proliferation in leaf development.

In *Arabidopsis*, loss-of-function mutants *tcp2, tcp4*, and *tcp10* caused slight phenotype defect. Meanwhile, *tcp2/4* double mutants exhibited an increased phenotype defect, and *tcp2/4/10* triple mutants showed the most significant phenotype defects, with increase in leaf size and signs of crinkling (Schommer et al., 2008). TCP2, 4, and 10 repeatedly regulated leaf development. *BrrTCP2* overexpression in *Arabidopsis* exhibited as small leaves with few epidermal cells. Overexpression of *BrrTCP2* in *tcp2/4/10* triple mutants restored the defect leaf phenotype to mimic wild-type leaf phenotype. *BrrTCP2* might function in leaf development via inhibiting cell proliferations.

## Conclusion

In summary, we identified 39 TCP genes, which were distributed on 10 chromosomes with different densities, in the turnip genome. Y2H analysis showed that these transcription factors preferentially formed heterodimers. Expression analysis showed that these genes exhibited varied expression profiles. In addition, BrrTCP2 was involved in leaf development via regulating cell proliferations.

## Author contributions

XS, YoY, and HS conceived and designed the study; JD, SH, QY, CW, and YuY performed the experiments and analyzed the data; XS, YoY, and JD wrote the paper; all authors have read and approved the final version.

### Conflict of interest statement

The authors declare that the research was conducted in the absence of any commercial or financial relationships that could be construed as a potential conflict of interest.
