# Supplementary material for: Genome-Wide Identification and Characterization of BrrTCP Transcription Factors in Brassica rapa ssp. rapa
Source: Front Plant Sci. 2017 Sep 12;8:1588. doi: 10.3389/fpls.2017.01588 (PMC5601045; doi:10.3389/fpls.2017.01588)
Supplement: Supplementary file 1 [file Table1.docx]

Table S1 qRT-PCR primers

| BrrTCP1 qF | TACCTTTCTCCACTCGCTGG |
| --- | --- |
| BrrTCP1 qR | TGATACCATTTGGCCCGGAG |
| BrrTCP1a qF | GGTTCGAGCCCCTATTCCTT |
| BrrTCP1a qR | GCTTTGGCTCTTGACCCCTT |
| BrrTCP1b qF | AGCATCTCCGGGTCAAATGG |
| BrrTCP1b qR | AAGACCTTGTGCCGTGTGAA |
| BrrTCP2 qF | GTGGCAAAGATCGACACAGC |
| BrrTCP2 qR | GGGCTGATCGTAGCCTAACC |
| BrrTCP3 qF | GCAAAGCTGTTGACTGGCTC |
| BrrTCP3 qR | GCAGCTTGTCGGATTGTGTC |
| BrrTCP4 qF | TGGGAGCAGCAGTCAATTCAG |
| BrrTCP4 qR | TCGGAGAACAAGGCGGGC |
| BrrTCP4a qF | CGACGTCGTTTCAGCCAGTA |
| BrrTCP4a qR | CTCACCTGAAGCAATTGGCG |
| BrrTCP4b qF | GAGTCGTTTTTCCCGGTGGT |
| BrrTCP4b qR | GCCATCGTACCCAAACGGAT |
| BrrTCP5 qF | CACAGTCCGTGGCCTTAGAG |
| BrrTCP5 qR | ACGACTTTGCTTGGCTGACT |
| BrrTCP6 qF | CGCGGTGGAAGAGTTAGGTT |
| BrrTCP6 qR | TTGAGAAACGGAGTCGGTGG |
| BrrTCP7 qF | CGGATCTTTAATCGAACACCAGC |
| BrrTCP7 qR | CCTCTTCCGTCGACTTTGCT |
| BrrTCP7a qF | GCAGCTGCCCTTTTTGTTCA |
| BrrTCP7a qR | CGTGTGTCATCCTCTCTCCG |
| BrrTCP7b qF | GCCTCACTCCGAGGAAACTC |
| BrrTCP7b qR | GTGTATCCTCGTCGGCTCTC |
| BrrTCP8 qF | AGAGGAGGTTCTCGCCATCT |
| BrrTCP8 qR | CTTTCGTGTGGCGGTCTTTC |
| BrrTCP9 qF | GTTAGCTTTTCCGGCTGCTC |
| BrrTCP9 qR | CTCGGAGCCATAACTGACGG |
| BrrTCP9a qF | TCCGTCAACGGAACGCTAAA |
| BrrTCP9a qR | AGGAAACCGAAACCGAGACC |
| BrrTCP10 qF | ACCCTCATCATCACGATGCC |
| BrrTCP10 qR | TCGAAATGAGACGTGGCGAA |
| BrrTCP12 qF | AAGATCTGCACCGCTCAAGG |
| BrrTCP12 qR | GCTCGCCTTATCGAAACCCA |
| BrrTCP13 qF | AGACGGTGGTGTGAACGAAG |
| BrrTCP13 qR | GCTCCACTCCAACTCGAAGA |
| BrrTCP13a qF | GCAAAGTGTGTACGTTGCGT |
| BrrTCP13a qR | GCTGCATTGAGCAACCAGTC |
| BrrTCP14 qF | CAGCGGTGGTCGTAGCTAAA |
| BrrTCP14 qR | GAGCTGGAACACCCTAGCTG |
| BrrTCP15 qF | ACTCCGACCCTAACGTTCTC |
| BrrTCP15 qR | AAGCCACTCGATGGTTTCAC |
| BrrTCP15a qF | CTGCTGCTCACCTTCGTACA |
| BrrTCP15a qR | TGTGACTCGTTCTTCGGACG |
| BrrTCP15b qF | ACCACAACCAGATGGGGAAC |
| BrrTCP15b qR | CCCCCGAATGATGAGGGTTG |
| BrrTCP17 qF | GAAAGTTGGGATCTCGGGGG |
| BrrTCP17 qR | AACTCTCTCGCGGTGTTGTT |
| BrrTCP17a qF | AGGGCTAAGTCAGCCTAGCA |
| BrrTCP17a qR | GGGTGAAAACCAGGTGGGAA |
| BrrTCP18 qF | GAGCAGCCTTTCTAACCCGT |
| BrrTCP18 qR | TGCTGCCTCTATCCTCGACT |
| BrrTCP18a qF | TCTTTTGAGGTGACGCCCTG |
| BrrTCP18a qR | TGGAAGTAGCTGCACCTTGG |
| BrrTCP19 qF | TGGGGAAAGCTTGGAAACATCT |
| BrrTCP19 qR | CCTGTTGCTTGTTTGGGTCG |
| BrrTCP20 qF | CCAAATTGGGGAGTTGGAGG |
| BrrTCP20 qR | ACTCTACCCTGAGCTTGACTC |
| BrrTCP20a qF | TGCTGTATCGAGCCACCATC |
| BrrTCP20a qR | ACCCCAATTTGGTCTCCCAC |
| BrrTCP20b qF | CGGTGAAACAATCCAGTGGC |
| BrrTCP20b qR | CAGTGAGAGACCCACCTTGG |
| BrrTCP21 qF | GTTTTCCAGCTGACGAGGGA |
| BrrTCP21 qR | AGAAGCGGTGGAGAAACTCG |
| BrrTCP21a qF | GGCGTCATAGTGGAGCATCT |
| BrrTCP21a qR | CTCTATGGTCTGGCCGTCTG |
| BrrTCP21b qF | AGAGAGCTCGGCCACAAATC |
| BrrTCP21b qR | AAGCGTTTTCCGAGAGCGAA |
| BrrTCP22 qF | CCGCGAAGTTGTTAAACGCA |
| BrrTCP22 qR | CTCCCACTTGGTTCCGACTC |
| BrrTCP23 qF | AGGATCGGTTCCAAGCACAG |
| BrrTCP23 qR | CCCTCGTGAGCTGGAAAACT |
| BrrTCP24 qF | CTGGAACAACCCATCTTCACG |
| BrrTCP24 qR | GCCATTCAACGGCTTTGCTG |
| BrrTCP24a qF | TAGGGTTTCACGAGCTACCG |
| BrrTCP24a qR | CAGAAGCCGCGTTGATAAGC |
| BrrACT2 qF | GGAATCCACGAGACGACTTAC |
| BrrACT2 qR | GCTCATACGGTCCGCAATAC |
