# Supplementary material for: Genome-Wide Identification and Characterization of BrrTCP Transcription Factors in Brassica rapa ssp. rapa
Source: Front Plant Sci. 2017 Sep 12;8:1588. doi: 10.3389/fpls.2017.01588 (PMC5601045; doi:10.3389/fpls.2017.01588)
Supplement: Supplementary file 2 [file Table2.docx]

Table S2 primers used for vector construction

| BrrTCP1F | CACCATGTCTTCTTCCAACAATGATTAC |
| --- | --- |
| BrrTCP1R | TGGGAACCCCCTGGGGAAACTC |
| BrrTCP1aF | CACCATGTCTTCTTCCAACAATGGTTAC |
| BrrTCP1aR | TGGGAACCCTCTGAAGAAATTTG |
| BrrTCP1bF | CACCATGTCTTCTTCCAACAATGATTAC |
| BrrTCP1bR | CGCTAGAGAAGAGTCTTGAACC |
| BrrTCP2F | CACCATGGTTATAGAGAAGCTTAGGAATG |
| BrrTCP2R | GTTCTTGCCTTTACCCTTTTGG |
| BrrTCP3F | CACCATGGCTGATGAAGAATCTAACC |
| BrrTCP3R | ATGTCGAGAATCAGATGAAGCAG |
| BrrTCP4F | CACCATGGCAGACGAAGCTCACAACTTTC |
| BrrTCP4R | ACGATGGCGAGAAATGGAGGAAG |
| BrrTCP4aF | CACCATGGCAGAAGACGACGAAGCTC |
| BrrTCP4aR | ATGGCGAGAAGCAGAGGACG |
| BrrTCP4bF | CACCATGGCAGACGACCACGACGAAG |
| BrrTCP4bR | ATGGCGAGAAACAGAGGAAGCAGAG |
| BrrTCP5F | CACCATGAGATCAAGAGAATGCGATG |
| BrrTCP5R | CGAATCTGAATCATTGTCATCAC |
| BrrTCP6F | CACCATGGATCCAAATCTCTTAAACCC |
| BrrTCP6R | CCCTTGTTTGTCTGCGTTCTCT |
| BrrTCP7F | CACCATGTCTAATAACGACGGAGTTATGATCT |
| BrrTCP7R | ACGCTGGTCATCCTCTCTCC |
| BrrTCP7aF | CACCATGCCAATCATATGCGCAGCAC |
| BrrTCP7aR | TGTGTCATCCTCTCTCCGACCCG |
| BrrTCP7bF | CACCATGTCTAACAACAACGACGGAGC |
| BrrTCP7bR | ACGCTGGTCATCATCCTCTCCC |
| BrrTCP8F | CACCATGGCAGACGAAGCTCACAACT |
| BrrTCP8R | CCCATCGCTATTTGAATTCT |
| BrrTCP9F | CACCATGGCGACGATTAAACACCACG |
| BrrTCP9R | GTGGTTCGATGATGACCGTGCT |
| BrrTCP9aF | CACCATGGCGACAATTAAGAAGCACG |
| BrrTCP9aR | GTGGTTCGATGACCGTGGTG |
| BrrTCP10F | CACCATGGGACTTAAAGGTTATAGCG |
| BrrTCP10R | GAGATGTGAGTTTGGAGGAGAAG |
| BrrTCP12F | CACCATGTTTCCTTCAATAGACACCAATGGC |
| BrrTCP12R | GTAGCAGAGATAATCGTATAGAG |
| BrrTCP13F | CACCATGGATACCGGCCCTTGGAA |
| BrrTCP13R | GACCGCCGCCGTCCTAGTT |
| BrrTCP13aF | CACCATGAATACCGGACCTTGGAGAGATG |
| BrrTCP13aR | CTTCCGGTCGAACTGGACCGA |
| BrrTCP14F | CACCATGGACGGCGGAGACAACG |
| BrrTCP14R | ATCTTGTGGATCTTCCTCACCGC |
| BrrTCP15F | CACCATGGATCCTGATCCAGATCATC |
| BrrTCP15R | GGAATGATGACTTGTGCTTCCATC |
| BrrTCP15aF | CACCATGCCGGCCATGTGCGCCGCAC |
| BrrTCP15aR | GGAATGATGACTTGTGCTTCCATCT |
| BrrTCP15bF | CACCATGCCTGCCATGTGCCCCGC |
| BrrTCP15bR | GGAATGATGACTCGTGCTTCCATCT |
| BrrTCP17F | CACCATGAGATCAAGAGACTGCGATGAAG |
| BrrTCP17R | TCTGTTGTTGCGATTAGCATTGG |
| BrrTCP17aF | CACCATGAGAACCAACTCAATGGGAAT |
| BrrTCP17aR | CTACTCGACATGATCTGTTTGCT |
| BrrTCP18F | CACCATGAACAACAGGTCTTTCAGTACTA |
| BrrTCP18R | CATATTATGGTAGTTGTACATGAGG |
| BrrTCP18aF | CACCATGAAAAATGCCAAGAAACCAAGC |
| BrrTCP18aR | CATATTATGATAGTTGTACAAGAAGTGATGAAC |
| BrrTCP19F | CACCATGGGGAAAGCTTGGAAACATC |
| BrrTCP19R | CGGAGATGTGTTGTCCCCTGAG |
| BrrTCP20F | CACCATGGTCATTGATCTAGATTCTCCTCC |
| BrrTCP20R | AAGCCCAGAGCCTTGAGAATCATC |
| BrrTCP20aF | CACCATGGATCCCAAGAACCCAAATCG |
| BrrTCP20aR | ACGCCCTGACCCTTGAGAATCATCT |
| BrrTCP20bF | CACCATGGATCCCAAGAACCCAAATC |
| BrrTCP20bR | TGAGCCTTGAGATTCATCTCTCC |
| BrrTCP21F | CACCATGTCGAACGACGACGGGACAG |
| BrrTCP21R | TGAGTTATCCTCCTCCTCCCTCCG |
| BrrTCP21aF | CACCATGACGAGCAACGACGGATCAG |
| BrrTCP21aR | TGGGTTATCCTCTTCCCTCCGA |
| BrrTCP21bF | CACCATGGAGAACAACGACGGAGCAATG |
| BrrTCP21bR | GCGTGAGTCATCCTCTTCTCTCCCG |
| BrrTCP22F | CACCATGGATCAGAACTCTCACGACC |
| BrrTCP22R | CTTTCACTTCTTGTCATCACCAC |
| BrrTCP23F | CACCATGGAAACTCGCACGGCGGAG |
| BrrTCP23R | CTTCTTGTCATCACCACCATTTTCAC |
| BrrTCP24F | CACCATGGAGGTCGACGACGACATTG |
| BrrTCP24R | TCTCCCATCCTTTCCTTTATCGTC |
| BrrTCP24aF | CACCATGGAAGACGACGACGACATTGAG |
| BrrTCP24aR | TCTTCTCTCCTTTCCTTTATCGTCG |
